# The Week in the Courts

**Published:** 1911-02-25

**Authors:** 


### The Week in the Courts

### ACTION AGAINST A BONESETTER.

In the King's Bench Division, before Mr. Justice Darling and a special jury, the hearing of an action has commenced by which Mr. C. R. Thomas seeks to obtain damages from Mr. H. A. Barker, bonesetter, of Park Lane, for alleged negligence in the performance of operations on the plaintiff's leg. The case was proceeding at the time of going to press.

### UNSUCCESSFUL CLAIM AGAINST A MEDICAL PRACTITIONER.

At the Bow County Court, before Judge Smyly, K.C., and a jury, a Mr. Knapp and his wife sought to recover damages from Mr. L. M. Dunlop, L.R.C.P., L.R.C.S. Edin., of Poplar, for alleged neglect. The plaintiff's case was that Mr. Dunlop was negligent in his treatment of two children, but the judge said plaintiff had produced no evidence whatever of neglect on the part of the defendant, and the jury having intimated that they had heard enough, judgment was given for the defendant with costs. Mr. Dunlop was represented by Mr. H. C. Dickens, instructed by the solicitors to the Medical Defence Union.

### ALLEGATIONS AGAINST A MEDICAL PRACTITIONER.

At an inquest held at Battersea on the body of a girl, aged five years, the father of the child alleged that the medical practitioner who was called in was intoxicated at the time of the visit. This allegation was denied by the practitioner, who explained that he had been very ill and his head was very bad. The jury returned a verdict of "Death from natural causes," but added a rider to the effect that they were not satisfied with the practitioner's conduct, and thought the attention of the General Medical Council should be called to the matter.

### MEDICAL PRACTITIONER'S LIBEL ACTION.

In the King's Bench Division of the High Court of Justice, before Mr. Justice Darling and a special jury, Mr. T. W. M. Harneis, M.R.C.S., L.R.C.P., sued the *Postman's Gazette* (the official organ of the Postmen's Federation) for an alleged libel contained in the issue of December 18, 1909. Mr. Harneis, who practises in Hackney, was in 1905 appointed medical officer of the Hackney Postal District. The paragraph complained of contained the following passages: "The present medical officer has not been over-zealous in giving attention to the men, as several have complained of inattention on his part. Furthermore, he seems to be labouring under the delusion that his position enables him to grossly insult those who are unfortunate enough to be compelled to go to him for medical treatment. We would advise all our members to take note of his actions in future, so that further steps may be taken with a view to bringing him to his senses and getting him to treat men properly." Mr. Harneis denied the imputations, and under cross-examination maintained that he invariably extended to every postman the same courtesy as to private patients. No complaints had ever been preferred against him. After the case had proceeded for some time, defendants' counsel announced that the defendants would apologise and withdraw their plea of justification, and would publish in the *Postal Gazette* and in two local papers an apology. Judgment was entered for plaintiff with 40s. nominal damages, the plaintiff's only object having been to clear his character.

